# Hydrogel-Mediated Local Delivery of Induced Nephron Progenitor Cell-Sourced Molecules as a Cell-Free Approach for Acute Kidney Injury

**DOI:** 10.3390/ijms251910615

**Published:** 2024-10-02

**Authors:** Kyoungmin Park, Wei-Wei Gao, Jie Zheng, Kyung Taek Oh, In-Yong Kim, Seungkwon You

**Affiliations:** 1 Department of Biotechnology, College of Life Sciences and Biotechnology, Korea University, Seoul 02841, Republic of Korea; skskrudals@korea.ac.kr (K.P.); gaoweiwei1502@163.com (W.-W.G.); devil88128@hotmail.com (J.Z.); ken2013@korea.ac.kr (K.T.O.); 2 Catholic High-Performance Cell Therapy Center & Department of Medical Life Science, College of Medicine, The Catholic University of Korea, Seoul 06591, Republic of Korea; 3 Institute of Animal Molecular Biotechnology, Korea University, Seoul 02841, Republic of Korea

**Keywords:** induced nephron progenitor cells, injectable hydrogel, cell-free regenerative medicine

## Abstract

Acute kidney injury (AKI) constitutes a severe condition characterized by a sudden decrease in kidney function. Utilizing lineage-restricted stem/progenitor cells, directly reprogrammed from somatic cells, is a promising therapeutic option in personalized medicine for serious and incurable diseases such as AKI. The present study describes the therapeutic potential of induced nephron progenitor cell-sourced molecules (iNPC-SMs) as a cell-free strategy against cisplatin (CP)-induced nephrotoxicity, employing hyaluronic acid (HA) hydrogel-mediated local delivery to minimize systemic leakage and degradation. iNPC-SMs exhibited anti-apoptotic effects on HK-2 cells by inhibiting CP-induced ROS generation. Additionally, the localized biodistribution facilitated by hydrogel-mediated iNPC-SM delivery contributed to enhanced renal function, anti-inflammatory response, and renal regeneration in AKI mice. This study could serve as a ‘proof of concept’ for injectable hydrogel-mediated iNPC-SM delivery in AKI and as a model for further exploration of the development of cell-free regenerative medicine strategies.

## 1. Introduction

Acute kidney injury (AKI) is a severe complication characterized by a sudden and often reversible reduction in kidney function, identified by a rapid increase in serum creatinine, a reduction in urine output, or both, regardless of pre-existing chronic kidney disease (CKD) [1]. This condition represents a global public health concern due to its high morbidity, mortality rates, and extensive healthcare costs, affecting more than 750 million of the global population, 10–15% of hospitalized patients and occasionally exceeding 50% among patients in the intensive care unit (ICU) [2,3]. Despite the high incidence and prevalence of AKI requiring hemodialysis and kidney replacement therapy, there are currently no established treatments or definitive clinical management guidelines [4,5]. Given the limited lifespan of dialysis and the scarcity of donor kidneys, exploring novel therapeutic avenues to potentially cure, prevent, or reverse kidney diseases becomes essential. Stem cell-mediated regenerative medicine emerges as an attractive approach under these circumstances.

Over the past decade, considerable attention has been directed towards stem cell transplantation as a potential therapeutic option for conditions where conventional medical interventions have shown limited efficacy. Various stem cell types, such as adult stem cells, embryonic stem cells, and induced pluripotent stem cells (iPSCs) have been transplanted in efforts for tissue repair or regeneration [6,7]. Notably, the emergence of iPSCs has generated tremendous enthusiasm because of their distinctive properties of infinite self-renewal and differentiation into cell types representing the three embryonic germ layers, thus enabling personalized medicine. However, persistent concerns regarding tumorigenicity hinder their clinical feasibility [8]. Furthermore, despite their impressive therapeutic potential demonstrated in preclinical animal models, clinical trials have produced inconsistent and highly variable results, mainly attributed to low rates of survival, engraftment, and persistence at the targeted transplant site [9,10]. Lineage-restricted progenitors, directly reprogrammed from somatic cells, present an alternative to iPSCs for incurable diseases. By reducing tumorigenic risks through bypassing pluripotency acquisition, they can more efficiently engraft and differentiate into desirable cell types at the injured site after transplantation [11]. This approach presents significant advantages compared to iPSCs including enhanced karyotypic stability, homogeneity, reduced tumorigenicity, and savings in both time and costs [12,13]. Lineage-restricted progenitors, generated by direct reprogramming, are therefore expected to be more widely applied as a complementary tool to the iPSC technique in personalized therapy for devastating diseases including AKI and CKD.

Meanwhile, the early hypothesis suggested that transplanted stem cells would differentiate into local phenotype cells, replacing damaged tissues during repair or regeneration. However, there is scarce evidence supporting this regeneration scenario. It gradually became apparent that the regeneration response depends rather on paracrine signaling communications between the transplanted cells and host cells through numerous biomolecules including growth factors, cytokines, and vesicles [14,15]. Instead of the direct use of living or infinitely proliferating cells, injured tissues can be mediated by invoking intrinsic repair mechanisms using stem cell-sourced molecules. Such an approach may address the limitations and concerns associated with stem cell transplantation by utilizing secreted molecules as a safer, controlled regenerative medicine strategy. Importantly, considering that renal progenitors disappear before birth due to their niche loss, the kidney lacks the capacity to generate new nephrons in response to injury [16], such stem cell-sourced molecules could be utilized as a biological control strategy for providing the paracrine cues that stimulate inherent regenerative capacities and trigger tissue repair. Indeed, the adult kidney exhibits a resilient capability for epithelial turnover through transiently adopting the phenotype of progenitors with reparative properties, achieved through injury-induced dedifferentiation of surviving resident cells. These cells contribute to the repair process by engaging in autocrine, endocrine, and/or paracrine signaling [17,18]. Taken together, renal progenitors generated from somatic donor cells can be appropriate as a cell source to produce therapeutic agents for personalized treatments in kidney diseases.

**Figure 1:**
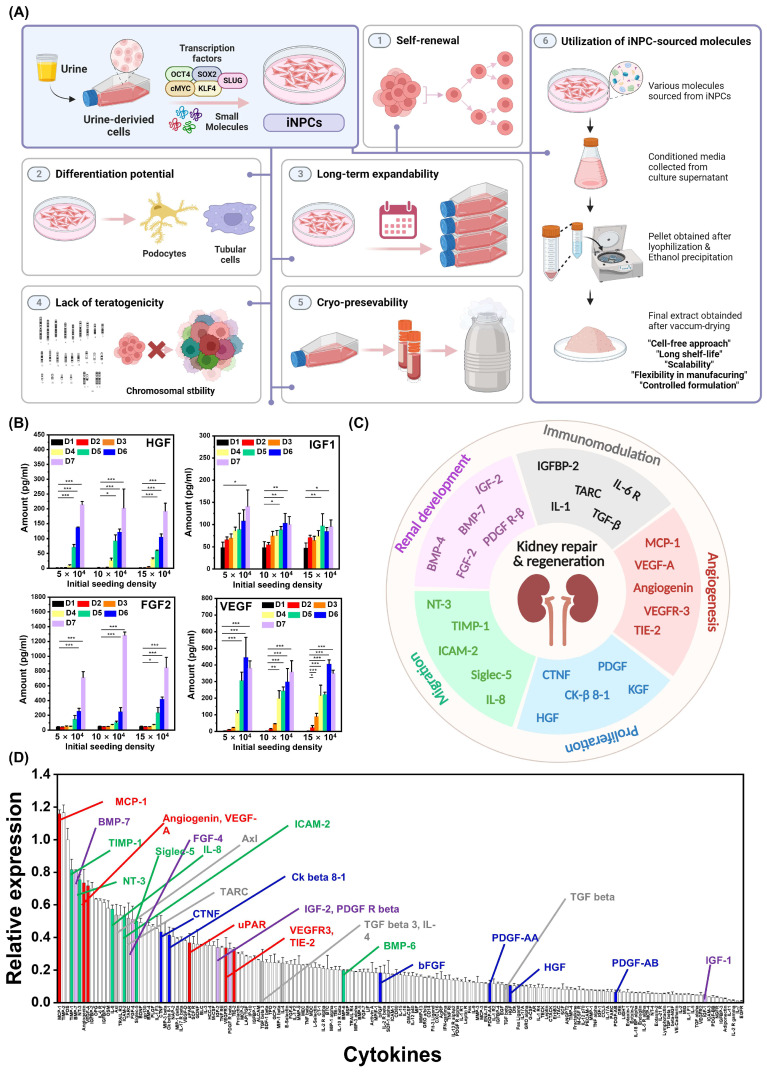
Preparation and profiling of iNPC-SMs. (**A**) Schematic representing the beneficial characteristics of iNPCs generated from urine-derived cells and the preparation process of iNPC-SMs as a therapeutic agent for renal regeneration. (**B**) ELISA analysis of paracrine factors (HGF, IGF1, FGF2, and VEGF) involved majorly in renal diseases and repair process, secreted from iNPCs in a cell density- and time-dependent manner. * *p* < 0.05, ** *p* < 0.01, *** *p* < 0.001. (**C**,**D**) Antibody-based protein array of iNPC-SMs including growth factors and cytokines. The array assessed proteins associated with immunomodulation, angiogenesis, proliferation, migration, and renal development, with potential implications for renal regeneration and recovery. Blot density was analyzed using ImageJ. Data are presented as mean ± SD from three independent experiments.

We have previously introduced a direct reprogramming approach for generating induced nephron progenitor cells (iNPCs) from human urine cells (hUCs) by inducing the enforced expression of OCT4, SOX2, KLF4, c-MYC, and SLUG, exposed to a specific combination of defined molecules (Figure 1A) [19,20]. The established iNPCs showed a self-renewal capacity, differentiation potential into kidney-lineage cells, long-term expandability under serum-free conditions, normal karyotype with a lack of teratogenicity, and cryo-preservation. Subsequently, when locally transplanted into a diabetic nephropathy (DN) mouse model, the iNPCs, despite showing a limited lifespan, prompted substantial improvements in various renal parameters, including glomerular hypertrophy, tubulointerstitial fibrosis, and reduced serum markers of kidney dysfunction [20]. This phenomenon is similar to the observation that transplanted mesenchymal stem/stromal cells exhibited low rates of survival at the targeted site [9,21]. Building upon these findings, the present study describes the therapeutic performance of locally released iNPC-sourced molecules (iNPC-SMs) as a cell-free strategy against cisplatin (CP)-induced nephrotoxicity, widely accepted as a model for AKI. These iNPC-SMs were prepared by ethanol precipitation, which is commonly used for the salting-out and purification of biological macromolecules, in a time- and cost-efficient manner [22,23]. This formulation strategy offers benefits in terms of simplicity, scalability, reproducibility, storage stability, long shelf life, and dose controllability. In addition, to ensure sustained delivery of iNPC-SMs, we employed an injectable and biodegradable hyaluronic acid (HA)-based hydrogel system, aiming to minimize systemic leakage and degradation. Overall, our findings suggest that hydrogel-mediated local delivery of iNPC-SMs deserves substantial attention as a promising cell-free regenerative medicine strategy for kidney diseases.

## 2. Results and Discussion

### 2.1. Preparation and Profiling of iNPC-SMs

Although the adult kidney has been traditionally regarded as a non-regenerative organ, it possesses a robust capacity for epithelial turnover in nephrons. After renal injury, membrane receptor density is elevated to facilitate the recruitment of external growth factors in anticipation of the repair and regeneration processes; the kidney itself is not the source of growth factors for the repairing proliferation [24]. Many studies have reported that renal regeneration occurs through the intricate molecular dynamics of various signaling pathways [25]. Similarly, our previous results demonstrated significant improvements in various renal parameters, despite the limited lifespan of the transplanted iNPCs. This is in line with the well-documented paracrine effects observed with MSCs, where transplanted cells, despite their low survival rates, exert therapeutic benefits primarily through the release of extracellular vesicles, cytokines, and growth factors [12,21]. Therefore, to optimize the procedure of collecting iNPC-sourced conditioned medium in a cell density- and time-dependent manner, we initially quantified iNPC-sourced paracrine factors. Notably, paracrine factors such as HGF, IGF1, FGF2 and VEGF have been robustly recognized in studies related to renal diseases and repair process (Figure 1B) [25]. These findings indicate that the productivity of representative therapeutic factors secreted from iNPCs is significantly influenced by time rather than cell density. As part of the strategy for localized and sustained delivery of iNPC-SMs using an injectable hydrogel system, ethanol precipitation was employed to desalt and concentrate the bioactive molecules in iNPC-conditioned medium. The ethanol precipitation-based approach for iNPC-SMs offers several potential advantages over traditional cell-free therapies, including simplicity, scalability, safety, and the ability to control dosage formulation [26]. The resulting iNPC-SMs appeared as a white powder, readily dissolvable in typical culture media up to a concentration of 1000 μg/mL. Profiling a broad spectrum of 174 human cytokines from iNPC-SMs, including growth factors and cytokines linked to immunomodulation, angiogenesis, proliferation, migration, and renal development, was accomplished through a human cytokine array (Figure 1C and Figure S1). Relative protein amounts were plotted accordingly (Figure 1D), where we specifically identified well-known key factors related to kidney repair and regeneration, including BMPs, FGFs, IGFs, VEGFs, and PDGFs. These findings collectively suggest that post-ethanol precipitation, the produced iNPC-SMs contained numerous bioactive molecules with the potential to induce the turnover of resident renal cells and consequently, initiate the regeneration process [27].

### 2.2. In Vitro Protective Effects of iNPC-SMs against CP-Induced Cytotoxicity

**Figure 2:**
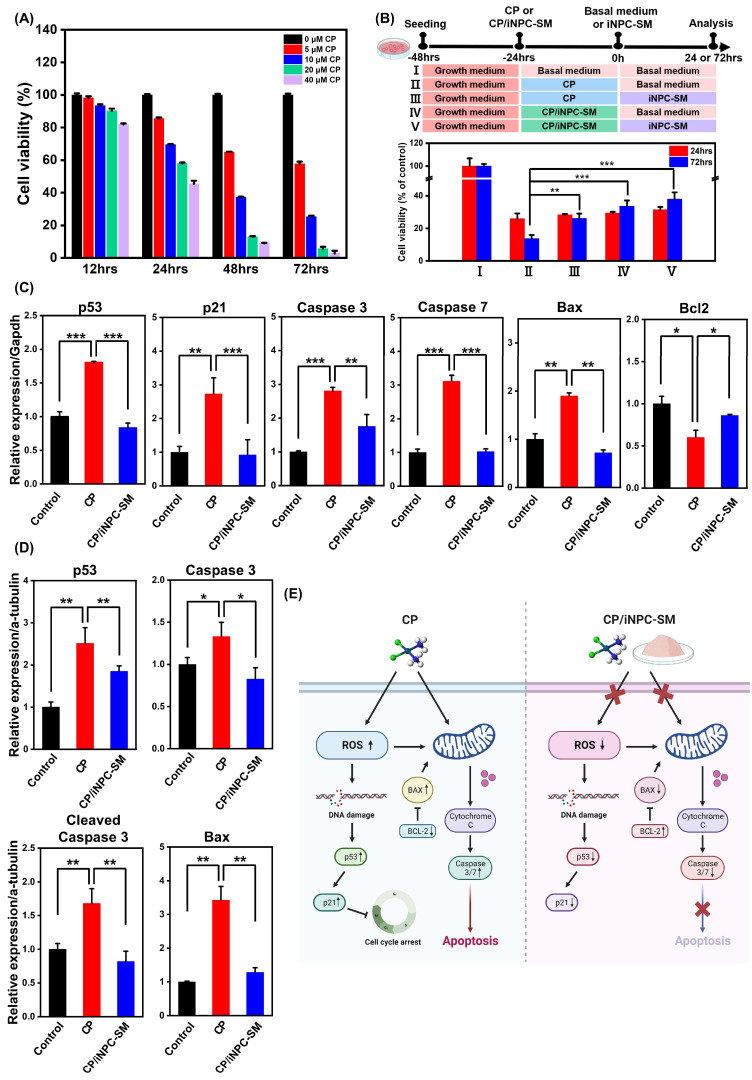
In vitro protective effects of iNPC-SMs against CP-induced cytotoxicity. (**A**) Time-dependent changes in viability of HK2 cells with various concentrations of CP. (**B**) Cell viability of HK2 cells exposed to different culture conditions supplemented with CP and iNPC-SM for 24 or 72 h. Cells were seeded at a density of 5 × 10^3^ cells per well in a 96-well plate. Viability was analyzed using a CCK -8 assay. (**C**) Real-time PCR analysis of cell arrest- and apoptosis-associated genes (p53, p21, Caspase 3/7, Bax, and Bcl2) in HK2 cells exposed to CP and CP/iNPC-SM. (**D**) Western blot analysis of apoptotic markers (p53, Caspase 3, Cleaved Caspase 3, and Bax) in HK2 cells exposed to basal medium, CP, and CP/iNPC-SM. Blot density was analyzed using ImageJ. Data are represented as mean ± SD. * *p* < 0.05, ** *p* < 0.01, *** *p* < 0.001. (**E**) Schematic illustration depicting the anti-apoptotic effect of iNPC-SMs via inhibiting ROS generation on CP-induced cytotoxicity.

Prior to investigating the biological functions of iNPC-SMs, we first evaluated CP-induced cytotoxicity in HK-2 cells, based on the previously described conditions [28]. Figure 2A shows a dose- and exposure time-dependent decrease in viability of HK-2 cells exposed to CP, as measured by the CCK-8 assay. The cells exposed to CP at concentrations of 20 and 40 μM for 48 and 72 h underwent significantly increased cell death. Thus, 10 µM of CP dose was chosen as the optimal concentration for subsequent studies. This observation was supported by visualizing the viability of HK-2 cells in the presence and absence of CP using live/dead staining (Figure S2A). Next, to evaluate the iNPC-SM-mediated protection or recovery in the presence of CP, these cells were subjected to five different culture conditions: basal medium (baM)+baM, CP+baM, CP+iNPC-SM, CP/iNPC-SM+baM, and CP/iNPC-SM+iNPC-SM (Figure 2B). After 24 h of CP exposure, the viability of HK-2 cells drastically decreased to approximately 20% and 10% at 24 and 72 h, respectively, indicating irrecoverable damage due to CP-induced toxicity. Despite CP causing extreme damage to the HK-2 cells, iNPC-SMs significantly enhanced cell survival at 48 h post-exposure. Importantly, co-treatment with iNPC-SMs during initial CP exposure resulted in higher cell survivability, implying their protective or recovery effects against CP-induced cytotoxicity.

Following renal epithelium injury, inflammatory mediators and heightened oxidative stress serve as formidable barriers to the repair and regeneration process, crucially contributing to renal injury expansion and posing risks for various complications in kidney disease patients [29]. This injury stems from nephrotoxic processes, including excessive intracellular calcium accumulation, metabolic disturbances, and mitochondrial dysfunction. Subsequently, this cascade of events culminates in the generation of ROS [30]. Previous studies indicate that once CP undergoes conversion into a reactive form, it readily interacts with intracellular antioxidants such as glutathione (GSH) [31]. The resulting dysfunction of GSH and associated antioxidants culminates in the accumulation of ROS within cells, triggering renal cell death. Additionally, ROS accelerate the fibrotic process through the initiation of inflammatory responses, initiating a feedback cycle that perpetuates inflammation and oxidative stress, thereby amplifying ROS levels. This cycle ultimately impairs regenerative capacity in injured tissues. In the present study, ROS generation in HK-2 cells exposed to CP and CP/iNPC-SM was assessed using DHE staining (Figure S2B). The addition of iNPC-SMs led to a great reduction in ROS generation, comparable to the control. Furthermore, we evaluated the expressions of cell arrest- and apoptosis-associated genes p53, p21, Caspase 3/7, Bax, and Bcl2. Upon iNPC-SMs addition, HK-2 cells exhibited significantly reduced mRNA expression of p53, p21, Caspase 3/7, and Bax while elevating the anti-apoptotic Bcl2 expression (Figure 2C). Corresponding proteins, such as p53, Caspase 3, Cleaved Caspase 3, and Bax, showed increased levels with iNPC-SMs addition (Figure 2D and Figure S2C). These results collectively support the substantial contribution of iNPC-SMs in enhancing cell survival by inhibiting CP-induced ROS generation (Figure 2E).

### 2.3. Preparation and Characterization of HA-Based Injectable Hydrogels

**Figure 3:**
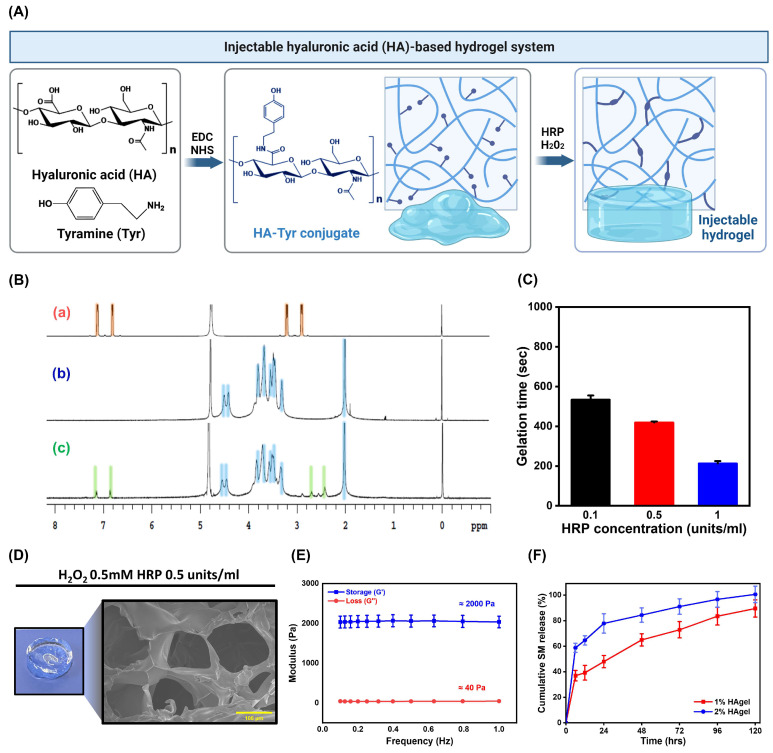
Preparation and characterization of hyaluronic acid-based injectable hydrogel (HAgel). (**A**) Schematic representation for the synthesis of HA-Tyr conjugate and the preparation process of HAgel. (**B**) ^1^H NMR spectrum of tyramine (a), hyaluronic acid (b), and HA-Tyr conjugate (c) dissolved in D_2_O. (**C**) Gelation times of HAgel at various concentrations of HRP and H_2_O_2_. (**D**) Digital image and scanning electron microscopic (SEM) image of HAgel. Scale bars = 100 μm. (**E**) Mechanical properties (storage modulus: G’, loss modulus: G”) of HAgel in the presence of HRP and H_2_O_2_. (**F**) Cumulative release profile of iNPC-SMs from HAgel in PBS at various concentrations of HA-Tyr conjugate. Data are represented as mean ± SD.

In view of clinical applicability and feasibility, our primary focus was on efficiently delivering natural biomacromolecules secreted by iNPCs to a given site, prioritizing practicality over the implementation of complex multifunctional systems. Here, we employed an injectable hydrogel entrapping iNPC-SMs within a semipermeable network comprising HA, minimizing their leakage, and protecting them against enzymatic degradation and immune response. The injectable system serves as a drug depot, gelling according to the surrounding environment, thereby facilitating sustained delivery of iNPC-SMs over an extended period. HA has been extensively explored for various regenerative medicine and tissue engineering purposes [32]. Related studies indicate its pivotal role in fostering the niche environment essential for renal branching morphogenesis and organogenesis [33]. Furthermore, HA is found ubiquitously distributed throughout the interstitial space of the developing kidney [34]. In this study, enzymatically cross-linkable HA was successfully prepared by coupling its carboxylic groups with primary amine groups of tyramine using EDC/NHS activation, through modifying as previously described [35], enabling gel formation in the presence of HRP and H_2_O_2_ (Figure 3A). The degree of substitution (DS) was determined by comparing the ratio of the relative peak integrations of the aromatic protons of tyramine (δ 6.8–7.2 ppm) and the methyl protons of HA (δ 2.0 ppm), using the ^1^H NMR spectrum (Figure 3B). The DS was 7.25 ± 0.25%. FTIR spectra of HA and HA-tyramine (HA-Tyr) showed peaks shown in Figure S3. The characteristic amide and carbohydrate-related bonds appear at 1680–1550 cm^−1^ and 1150–940 cm^−1^, respectively [36]. These results suggest that Tyr was successfully conjugated to HA via amide bond.

The gelation times of HA-based hydrogel (HAgel) could be controlled by mixing HRP (0.1, 0.5, 1.0 units/mL at final concentration) and 0.5 mM H_2_O_2_ immediately before gelation (Figure 3C). Figure 3D shows the digital and cross-sectional morphologies of HAgel prepared with 0.5 units/mL HRP and 0.5 mM H_2_O_2_. The mechanical property of HAgel was examined at 37 °C using a rheometer. Figure 3E shows that the storage modulus (G’) of the HAgel was approximately 2000 Pa, surpassing the loss modulus (G”) of approximately 40 Pa, a substantial difference that confirms successful gelation. Considering the storage modulus range of 2000–5000 Pa observed in healthy glomeruli [37] and previous findings demonstrating substrate stiffness-dependent regulation of proliferation, differentiation, and migration of renal progenitor cells [38], we selected a 1% (*w*/*v*) concentration of HA for this study. Next, we investigated the effect of 1 and 2% (*w*/*v*) HA concentrations on drug release from iNPC-SM-entrapped HAgel (SM-HAgel) using FITC-labeled iNPC-SMs. Figure 3F displays the in vitro release profiles of iNPC-SMs from SM-HAgel at the designed time points, 0, 6, 12, 24, 48, 72, 96 and 120 h. Whereas the release of iNPC-SMs was extended over 120 h, the cumulative release gap between hydrogels with 1 and 2% (*w*/*v*) HA concentrations gradually narrowed over time, suggesting that the drug release from HAgel depended on the polymer concentration and crosslinking density. Understanding the diffusion behavior of macromolecules within hydrogels is crucial for explaining their effectiveness as drug delivery systems or tissue engineering scaffolds. To enhance theoretical understanding and practical applications, further investigation into macromolecule diffusion could be conducted using techniques such as single-particle tracking (SPT), dynamic light scattering (DLS), and nuclear magnetic resonance (NMR) [39,40,41].

### 2.4. Hydrogel-Mediated Delivery of iNPC-SMs to AKI Mice

**Figure 4:**
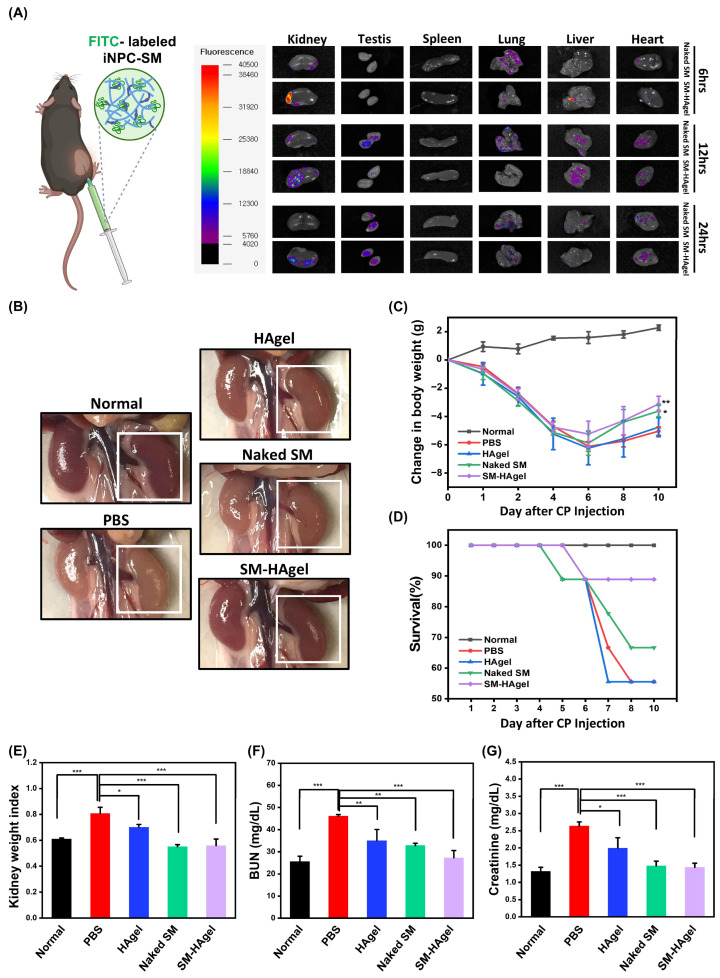
Hydrogel-mediated delivery of iNPC-SMs to AKI mice. (**A**) In vivo biodistribution of iNPC-SMs in the kidney, testis, spleen, lung, liver, and heart of C57B/6NTac mice injected with Naked SM and SM-HAgel. The SM-HAgel group exhibits a greater and sustained retention of iNPC-SM in the kidney from 6 to 24 h post-injection compared to the naked SM group. (**B**) Representative images of the kidneys on day 10 in normal mice and CP-treated mice injected with PBS, HAgel, Naked SM, and SM-HAgel. (**C**–**E**) Body weights (**C**), survival rates (**D**), and kidney weight (**E**) until day 10 of normal mice and CP-treated mice injected with PBS, HAgel, Naked SM, and SM-HAgel (n = 9). Significant differences in body weight changes compared to PBS are indicated. ** denotes significant differences for SM-HAgel, and * indicates significance for Naked SM. (**F**–**G**) Renal function analysis of BUN and creatinine in normal mice and CP-treated mice injected with PBS, HAgel, Naked SM, and SM-HAgel. Data are represented as mean ± SD. * *p* < 0.05, ** *p* < 0.01, *** *p* < 0.001.

An in vivo biodistribution study was conducted to monitor the fate of iNPC-SMs released from HAgel after injection. As shown in Figure 4A, both groups displayed a gradual decay of bioluminescence signals in major organs (kidney, testis, spleen, lung, liver, and heart) over time. Importantly, the bioluminescence signals at the kidney site in the SM-HAgel group appeared stronger at the same time point and persisted longer compared to that in the Naked SM group. These findings suggest the efficacy of hydrogel-mediated local delivery in maintaining and sustaining elevated levels of iNPC-SMs within the kidney. For in vivo evaluation of hydrogel-mediated delivery of iNPC-SMs in AKI, we locally injected PBS, HAgel, Naked SM, and SM-HAgel. At day 10 post-injection, kidneys in the PBS group displayed a rapid loss of color, implying glomerulosclerosis and tubulointerstitial fibrosis, whereas mice injected with other agents, particularly SM-HAgel, displayed suppressed color changes, suggesting potential renal function recovery (Figure 4B). These observations were supported by changes in body weight, survival rates, and kidney weight (Figure 4C–E). Moreover, the SM-HAgel group exhibited the lowest levels in blood urea nitrogen (BUN) and creatinine compared to the other groups (Figure 4F,G), highlighting the protective efficacy of injected iNPC-SMs against renal dysfunction in AKI mice. These findings suggest that renal function could be enhanced through indirect mechanisms via exogenous paracrine factors, similar to the direct use of progenitors.

### 2.5. The Therapeutic Actions of iNPC-SMs in AKI Mice

**Figure 5:**
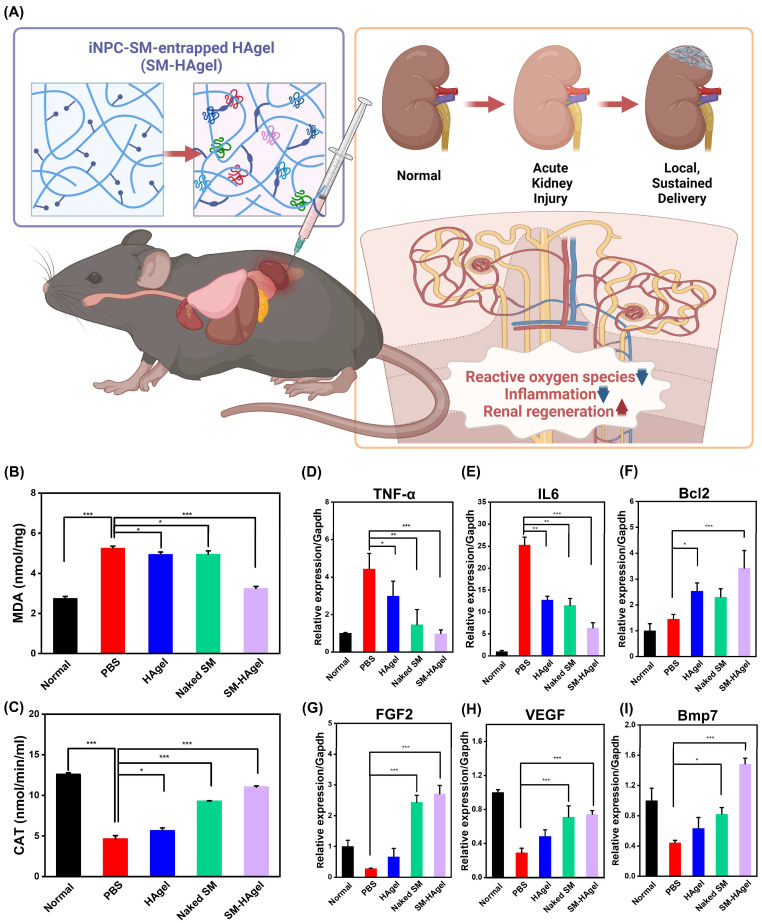
The therapeutic actions of iNPC-SMs in AKI mice. (**A**) Schematic representing the therapeutic actions of HAgel-mediated delivery of iNPC-SMs in acute kidney injury. (**B**,**C**) Quantitative analysis of pro-oxidant markers (malondialdehyde and MDA) and antioxidant enzymes (catalase and CAT) in normal and CP-treated mice injected with PBS, HAgel, Naked SM, and SM-HAgel. (**D**–**I**) Real-time PCR analysis of pro-inflammatory markers (TNF-α and IL6), anti-inflammatory marker (Bcl2), and the markers associated with renal regeneration (FGF2, VEGF, and Bmp7) in normal and CP-treated mice injected with PBS, HAgel, Naked SM, and SM-HAgel. Data are represented as mean ± SD. * *p* < 0.05, ** *p* < 0.01, *** *p* < 0.001.

Oxidative stress and inflammation interact in a mutually reinforcing manner, creating a self-propagating cascade that results in the gradual deterioration of target organs and dysfunction. The expression of the pro-oxidant marker, malondialdehyde (MDA), was highly decreased in the SM-HAgel group (3.25 ± 0.09 nmol/mg) compared to the PBS group (5.27 ± 0.09 nmol/mg) (*p* < 0.001). In contrast, the activity of antioxidant enzyme catalase (CAT) was markedly elevated in the SM-HAgel group (11.03 ± 0.13 nmol/min/mL) compared to the PBS group (4.62 ± 0.41 nmol/min/mL) (*p* < 0.001) (Figure 5B,C). The local inflammatory response to the injection of SM-HAgel in AKI mice was assessed by measuring mRNA expression of the pro-inflammatory cytokines TNF-α and IL6 and an anti-inflammatory marker Bcl2 (Figure 5D–F). When compared to the PBS group, The SM-HAgel group exhibited a noteworthy reduction in the expression of pro-inflammatory cytokines, accompanied by a substantial increase in Bcl2 expression (*p* < 0.001). Collectively, these results suggest that hydrogel-mediated local delivery of iNPC-SMs elicits antioxidant and anti-inflammatory effects on CP-induced nephrotoxicity.

To assess the impact of SM-HAgel injection on the molecular signals governing regeneration in AKI mice, we selected three indicators participating in renal regeneration (FGF2, VEGF, and Bmp7) (Figure 5G–I). Expression of these genes was significantly higher in the SM-HAgel group than in the PBS group (*p* < 0.001). After kidney injury, there is an elevation in FGF2 and VEGF expression within the kidney, contributing to the regulation of oxidative stress and the inflammatory response during the recovery phase [42]. Moreover, during kidney development, the expression of Bmp7 is crucial for determining nephron numbers and organ size. It acts on NPCs to maintain their population and responsiveness to differentiation cues. Notably, in adult kidneys, Bmp7 protects the kidney against injury and promotes regeneration by mitigating inflammation, apoptosis, and fibrosis [43]. Consequently, our results suggest that the injection of SM-HAgel provides molecular signals that trigger the initiation of renal regeneration following AKI through anti-inflammatory and antioxidant responses.

## 3. Materials and Methods

### 3.1. Ethics Statement

This study was approved by the Institutional Review Board of Korea University (No. -1040548-KU-IRB-18-62-A-2) and all experiments involving animals and humans were conducted according to the ethical policies and procedures approved by the Institutional Animal Care and Use Committee at Korea University (KUIACUC-2020-0028) and complied with the guidelines set by the National Research Council’s Guide for the Care and Use of Laboratory Animals.

### 3.2. Animals and Cells

Male C57B/6NTac mice aged 8–10 weeks weighing 20 to 25 g were used in this study. All mice were housed and fed in separate cages with a stable temperature and humidity under pathogen-free conditions. Immortalized human proximal tubular epithelial cells (HK-2) were obtained from the Korean cell line bank (KCLB). HK-2 cells were cultured in Roswell Park Memorial Institute (RPMI) 1640 medium (Hyclone, Logan, UT, USA) with 10% (*v*/*v*) fetal bovine serum (FBS; Hyclone) and 1% (*v*/*v*) penicillin/streptomycin (P/S; Lonza, Basel, Switzerland) under a humidified incubator with 5% CO_2_ at 37 °C. iNPCs used in this study were generated from hUCs as previously described [19]. Briefly, hUCs were seeded at a density of 5 × 10^4^ cells per well into a Matrigel-coated 6-well plate and incubated in UC proliferation medium for 2 days. Then, the hUCs transduced with TFs (OCT4, SOX2, KLF4, c-MYC, and SLUG) were exposed to a modifying nephron progenitor expansion medium (mNPEM) composed of RPMI 1640 supplemented with 100 ng/mL FGF9 (Peprotech, Rocky Hill, NJ, USA), 30 ng/mL BMP7 (Peprotech), 1.25 µM CHIR 99021 (R&D systems, Minneapolis, MN, USA), 125 nM LDN-193189 (Peprotech) and antibiotics under a humidified incubator with 5% CO_2_ in air at 37 °C. Formed iNPC colonies were picked as individual clones, selected, and expanded in a Matrigel-coated plate in mNPEM.

### 3.3. Preparation of iNPC-SMs

To prepare iNPC-SMs, iNPCs were seeded at a density of 5 × 10^5^ cells per plate (100 mm) and incubated in the mNPEM for 24 h. Subsequently, these cells were exposed to serum-free Advanced RPMI 1640 with 1% P/S and incubated for an additional 3 days. The culture medium conditioned by iNPCs was centrifuged at 1000 rpm for 10 min to eliminate cell debris and filtered using a 0.22 μm pore size filter. The resulting conditioned medium was aliquoted into 50 mL tubes, with 25 mL in each tube, followed by lyophilization. The freeze-dried powder was dissolved concentratedly in ultra-pure water, precipitated by adding cold ethanol (9-fold volume of the water volume), and placed at −20 °C overnight. After centrifuging at 4000 rpm for 30 min, the supernatant was carefully discarded. The resulting precipitates were then washed three times with cold ethanol, and the resulting iNPC-SMs were dried under vacuum to yield the product as a white powder.

### 3.4. ELISA

ELISA analysis was performed to detect the paracrine factors in the medium collected immediately after conditioning iNPCs in a cell density- and time-dependent manner. iNPCs were cultured at various cell densities in 6-well plates supplemented with mNPEM overnight. Subsequently, these cells were washed, and the medium was replaced with fresh mNPEM. After each culture condition, the resulting medium was collected. The expression levels of paracrine factors related to renal diseases and repair, including hepatocyte growth factor (HGF, #P14120), insulin-like growth factor 1 (IGF1, #P05019), basic fibroblast growth factor (FGF2, #P09038) and vascular endothelial growth factor (VEGF, #P15692) were measured using ELISA assay kits (all kits from RayBiotech, Norcross, GA, USA). Each factor was analyzed in triplicate according to the manufacturer’s instructions.

### 3.5. Cytokine Array

A human cytokine antibody array was performed to identify the cytokines present in iNPC-SMs by rehydrating after ethanol precipitation. All assays were carried out in triplicate according to the manufacturer’s instructions (RayBio^®^ C-Series Human Cytokine Antibody Array 6/7/8 Kit, RayBiotech, Norcross, GA, USA).

### 3.6. Cell Viability

Cell viability was evaluated using a Cell Counting Kit-8 (CCK-8, Dojindo Molecular Technologies, Rockville, MD, USA) and further determined by staining with Calcein/EthD (live/dead cytotoxicity kit, Invitrogen, Carlsbad, CA, USA). For the CCK-8 assay, 0.5 × 10^4^ HK-2 cells were initially seeded in a 96-well plate. The following day, each well was treated with 10 μL of CCK and incubated for 2 h at 37 °C. All assays were measured in quadruplicate using a microplate reader. The OD value was measured at 450 nm and cell viability was calculated using the following equation: Cell viability (%) = (OD value of each group − OD value of the blank)/(OD value of control − OD value of blank).

### 3.7. Reactive Oxygen Species Assay

The generation of reactive oxygen species (ROS) was determined by fluorescent staining with dihydroethidium (DHE) staining (DHE assay kit, Abcam, Cambridge, UK). The live cells were incubated in the presence of 5 µM DHE for 15 min and fixed in 4% paraformaldehyde (PFA) after PBS washing. Permeabilization was performed by 0.1% Triton X-100 for 10 min and nuclei were stained with DAPI for 5 min at room temperature, observed by a fluorescence microscope (IX71, OLYMPUS, Tokyo, Japan).

### 3.8. Real-Time PCR Analysis

Total RNAs were isolated from cells with a TRIzol (Thermo, Waltham, MA, USA)-mediated method. cDNAs were prepared by using RT PreMix (Bioneer, Daejeon, Republic of Korea) with oligo dT primer according to the manufacturer’s instructions. Amplification of gene fragments was performed by using 25 ng of cDNA, specific primers, and PCR PreMix (Bioneer) in a 20 µL total reaction volume. Real-time PCR was conducted with SYBR Green Supermix (Bio-Rad) using a CFX Connect™ Real-Time PCR Detection System (Bio-Rad, Hercules, CA, USA). The PCR analysis was performed in triplicate and the housekeeping gene GAPDH was used as an internal standard. The primer sequences are listed in Table S1.

### 3.9. Western Blot

Western blotting was performed in triplicate by re-suspending cells in cell lysis buffer with a protease inhibitor cocktail (Roche, Basel, Switzerland). RIPA buffer containing a protease inhibitor cocktail (Roche Molecular Diagnostics, Pleasanton, CA, USA) was used to extract proteins. After centrifuging at 12,000× *g* for 30 min at 4 °C, the concentrations of the protein lysates were determined using a Bradford assay kit (Bio-Rad, Hercules, CA, USA). Subsequently, the obtained proteins were separated through SDS-PAGE on a 4–12% gradient gel and then transferred onto a polyvinylidene difluoride membrane (EMD Millipore, Burlington, MA, USA). The resulting blots were blocked with Tris-buffered saline with 0.1% Tween 20 and 3% skim milk for 1 h and placed with primary antibodies at 4 °C overnight, followed by secondary antibody labeling with horseradish peroxidase (HRP)-conjugated IgG (1:1000, Abcam, Cambridge, MA, USA). The signals were detected using SuperSignal West Pico Chemiluminescent Substrate (Thermo, Waltham, MA, USA). The primary antibodies are listed in Table S2.

### 3.10. Synthesis of Hyaluronic Acid-Tyramine Conjugate

Hyaluronic acid-tyramine (HA-Tyr) conjugate was synthesized by coupling HA carboxylic groups with primary amine groups of tyramine using EDC/NHS activation, through modifying as previously described [44]. Briefly, HA (0.6 g, 1.5 mmol of COOH groups) was dissolved in 20 mL of PBS. EDC (0.6 g, 3.0 mmol) and NHS (0.3 g, 3.0 mmol) were added to activate the carboxyl group of HA. The HA solution was stirred for 2 h at room temperature. The NHS-activated HA was precipitated and washed with ethanol and diethyl ether. The resulting precipitate was dissolved in 18 mL of PBS, mixed with tyramine (0.4 g, 2.4 mmol) in 2.4 mL of DMF, and adjusted to pH 7.5. After stirring at room temperature overnight, the reaction mixture was precipitated and washed with ethanol and diethyl ether again. The final precipitate was dialyzed (MWCO 1000, RC dialysis tubing, Spectrum Labs, Gardena, CA, USA) against 0.1M NaCl for 2 days, a mixture of distilled water (DW) and ethanol (5:1) for 1 day, and DW for 1 day. The HA-Tyr conjugate was obtained as a solid powder after lyophilization. The degree of substitution was calculated from ^1^H NMR measurements (Varian NMR Systems 500 MHZ, Varian Inc., Palo Alto, CA, USA) by comparing the ratio of the relative peak integrations of the aromatic protons of tyramine (δ 6.8–7.2 ppm) and the methyl protons of HA (δ 2.0 ppm). The substitution degree was 7.25 ± 0.25%. The FT-IR data of hyaluronic acid, tyramine and conjugate were measured using Cart 630 FT-IR (Agilent).

### 3.11. Preparation and Characterization of HA-Hydrogel

The HA-Tyr conjugate was completely dissolved in PBS, sterile filtered and diluted to the designed concentrations. HA-hydrogels (HAgel) with or without iNPC-SMs were prepared by adding HRP and H_2_O_2_ solutions. The cross-sectional morphology of HAgel was observed using scanning electron microscopy (SEM) (Quanta 250 FEG, FEI, Hillsboro, OR, USA). The SEM samples were prepared by cross-sectioning following immersion in liquid nitrogen. The gelation times were investigated using a vial inversion method following the mixing of H_2_O_2_ (0.5 mM at a final concentration) and HRP (0.1, 0.5, 1.0 units/mL at a final concentration). The absence of flow upon vial inversion was interpreted to be indicative of a gel-like state. The mechanical properties of hydrogels were measured using an MCR 301 rheometer (Anton Paar, Graz, Austria) using a 25 nm diameter parallel plate at 37 °C. The hydrogels were prepared in a mold with a 20 mm diameter and a height of 5 mm. The initial force of the rheometer is 0.5 N/m^2^ and the storage modulus (G’) was recorded using a strain of 1% and a frequency from 0 to 1 Hz. In vitro release profiling of iNPC-SM from HAgel was measured by fluorescence spectrometer using an FITC-labeled iNPC-SM. The HA-Tyr conjugate was dissolved in PBS at concentrations of 1 or 2% (*w*/*v*), followed by dissolving FITC-labeled iNPC-SMs (2 mg/mL at final concentration) at 4 °C overnight with gentle agitation. The resulting mixture was aliquoted into a 96-well plate, with 100 μL added to each well. HAgel with FITC-labeled iNPC-SMs was subsequently prepared by mixing HRP and H_2_O_2_ solutions. After preparation, 100 μL of PBS was added to each well, and at the designed time points (0, 6, 12, 24, 48, 72, 96 and 120 h), the supernatant was collected and replaced with fresh PBS. The measurements were conducted in triplicate using a microplate reader.

### 3.12. Biodistribution Study

For the biodistribution study, C57B/6NTac mice were obtained from Dae Han Bio Link (Seoul, Republic of Korea). To visualize the retention and distribution of iNPC-SM within the kidneys and other organs, FITC-labeled iNPC-SMs were prepared. After anesthesia, iNPC-SMs were rehydrated in PBS and iNPC-SM-entrapped HAgel (SM-HAgel) were locally injected into the kidneys in a volume of 50 μL (2 μg/μL for iNPC-SM, 10 μg/μL for HA-Tyr). For the preparation of SM-HAgel, H_2_O_2_ (0.5 mM at a final concentration) and HRP (0.1 units/mL at a final concentration) were used. At time points of 6, 12, and 24 h, the mice were euthanized, and the organs (kidney, testis, spleen, lung, liver, and heart) were excised for fluorescence imaging. Quantification of iNPC-SM was investigated using a FOBI fluorescence imaging system (NeoScience Co., Ltd., Seoul, Republic of Korea) by fixing blue channels for imaging.

### 3.13. In Vivo Delivery and Renal Recovery

For in vivo renal recovery evaluation, mice were randomly divided into five groups (9 mice/group): Normal, PBS, HAgel (10 μg/μL), Naked SM (2 μg/μL), SM-HAgel (12 μg/μL). Before injecting the formulations, CP was pre-treated to all the groups, except the normal group, to establish the AKI model. After 24 h, each group was treated, and body weight changes and survival rates were recorded until day 10. Subsequently, blood samples and kidney tissues were collected. To assess renal function, serum samples were obtained by centrifuging at 14,000 rpm for 5 min, and the values of BUN and creatinine were determined using a BUN kit (Roche, Basel, Switzerland) and a creatinine kit (R&D systems, Minneapolis, MN, USA), following the manufacturer’s instructions. Oxidative stress (MDA and CAT) was assessed using commercially available kits (BioVision, Exton, PA, USA). Pro-inflammatory cytokines, TNF-α and IL6, and the anti-inflammatory marker Bcl2 were determined using real-time PCR. Additionally, renal regeneration and its underlying molecular signals were assessed by analyzing the expression of renal regeneration-associated markers (FGF2, VEGF, and Bmp7). At least three independent samples were analyzed.

### 3.14. Statistical Analysis

All data are expressed as mean values ± SD in n replicates. Changes in variables were analyzed using ANOVA with Tukey’s post hoc test and a paired two-tailed Student’s *t* test for multiple comparisons. Differences between samples were considered to be significant at * *p* < 0.05, ** *p* < 0.01, or *** *p* < 0.001.

## 4. Conclusions

With pioneer transcription factors OCT4, SOX2, KLF4, c-MYC, and SLUG orchestrating the reprogramming process, we previously proposed a novel method to generate iNPCs from hUCs. These cells, comparable to ESC-derived NPCs, offer long-term expandability in a serum-free condition, teratogenicity absence, and cryo-preservability. Despite their lineage similarity, high integration into the kidney, and significant therapeutic efficacy, hUC-derived iNPCs exhibited a limited lifespan, providing clues for the translation of kidney-specific iNPC-SMs into a therapeutically valuable agent. In contrast to stem cell transplantation, a cell-free approach utilizing stem cell-sourced bioproducts offers several advantages for clinical applications. This approach alleviates numerous safety concerns associated with administered cells, including tumorigenicity, emboli formation, infection transmission, and storage management. Furthermore, the iNPC-SMs, obtained through a straightforward ethanol precipitation method, provide several benefits such as high yield and scalable production, controllable formulation, and flexibility in fabrication techniques. These advantages contribute to economic and clinical feasibility, making them comparable to other cell-free approaches using exosomes or decellularized materials. The present ‘proof-of-concept’ study showed that iNPC-SMs exhibit the protective or recovery effects on HK-2 cells through inhibiting CP-induced ROS generation. In addition, the hydrogel-mediated delivery of iNPC-SMs led to localized biodistribution and significant therapeutic efficacy in a mouse model of AKI. Further studies are necessary to fully understand the therapeutic potential of iNPC-SMs in a time- and efficacy-dependent manner, which will help optimize the administration timing, dosage, and frequency of iNPC-SM-containing HAgel in clinical settings. Furthermore, the present study focused on elucidating the functions or paracrine actions of iNPCs. Further investigation and in-depth exploration of the optimization of the HAgel system for iNPCs are also needed to minimize inconsistencies between research and clinical outcomes. Our findings suggest that this cell-free strategy could serve as a promising therapeutic approach for kidney diseases and can also be expanded to target various diseases through bioactive molecules with specific properties derived from functional cell sources across diverse tissues and organs.

## Data Availability

Data will be available on request.

## Supplementary Materials

The following supporting information can be downloaded at: https://www.mdpi.com/article/10.3390/ijms251910615/s1.

## References

[1] Bonventre J.V., Yang L. (2011). Cellular pathophysiology of ischemic acute kidney injury. J. Clin. Investig..

[2] Ronco C., Bellomo R., Kellum J.A. (2019). Acute kidney injury. Lancet.

[3] Thurlow J.S., Joshi M., Yan G., Norris K.C., Agodoa L.Y., Yuan C.M., Nee R. (2021). Global Epidemiology of End-Stage Kidney Disease and Disparities in Kidney Replacement Therapy. Am. J. Nephrol..

[4] Tolwani A. (2012). Continuous Renal-Replacement Therapy for Acute Kidney Injury. N. Engl. J. Med..

[5] Benoit S.W., Devarajan P. (2018). Acute kidney injury: Emerging pharmacotherapies in current clinical trials. Pediatr. Nephrol..

[6] Zakrzewski W., Dobrzyński M., Szymonowicz M., Rybak Z. (2019). Stem cells: Past, present, and future. Stem Cell Res. Ther..

[7] Margiana R., Markov A., Zekiy A.O., Hamza M.U., Al-Dabbagh K.A., Al-Zubaidi S.H., Hameed N.M., Ahmad I., Sivaraman R., Kzar H.H. (2022). Clinical application of mesenchymal stem cell in regenerative medicine: A narrative review. Stem Cell Res. Ther..

[8] Yamanaka S. (2020). Pluripotent Stem Cell-Based Cell Therapy-Promise and Challenges. Cell Stem Cell.

[9] Hoang D.M., Pham P.T., Bach T.Q., Ngo A.T.L., Nguyen Q.T., Phan T.T.K., Nguyen G.H., Le P.T.T., Hoang V.T., Forsyth N.R. (2022). Stem cell-based therapy for human diseases. Signal Transduct. Target. Ther..

[10] Li Y., Hao J., Hu Z., Yang Y.-G., Zhou Q., Sun L., Wu J. (2022). Current status of clinical trials assessing mesenchymal stem cell therapy for graft versus host disease: A systematic review. Stem Cell Res. Ther..

[11] Fox I.J., Daley G.Q., Goldman S.A., Huard J., Kamp T.J., Trucco M. (2014). Stem cell therapy. Use of differentiated pluripotent stem cells as replacement therapy for treating disease. Science.

[12] Tanabe K., Haag D., Wernig M. (2015). Direct somatic lineage conversion. Philos. Trans. R. Soc. B Biol. Sci..

[13] Wang H., Yang Y., Liu J., Qian L. (2021). Direct cell reprogramming: Approaches, mechanisms and progress. Nat. Rev. Mol. Cell Biol..

[14] Sart S., Jeske R., Chen X., Ma T., Li Y. (2020). Engineering Stem Cell-Derived Extracellular Matrices: Decellularization, Characterization, and Biological Function. Tissue Eng. Part B Rev..

[15] O’Brien T., Barry F.P. (2009). Stem Cell Therapy and Regenerative Medicine. Mayo Clin. Proc..

[16] Hartman H.A., Lai H.L., Patterson L.T. (2007). Cessation of renal morphogenesis in mice. Dev. Biol..

[17] Kusaba T., Lalli M., Kramann R., Kobayashi A., Humphreys B.D. (2014). Differentiated kidney epithelial cells repair injured proximal tubule. Proc. Natl. Acad. Sci. USA.

[18] Berger K., Bangen J.M., Hammerich L., Liedtke C., Floege J., Smeets B., Moeller M.J. (2014). Origin of regenerating tubular cells after acute kidney injury. Proc. Natl. Acad. Sci. USA.

[19] Gao W.W., Zheng J., Yun W., Kang P.J., Park G., Song G., Kim I.Y., You S. (2021). Generation of induced nephron progenitor-like cells from human urine-derived cells. Int. J. Mol. Sci..

[20] Gao W.-W., Chun S.Y., Kim B.S., Ha Y.-S., Lee J.N., Lee E.H., Kim I.Y., You S., Kwon T.G. (2022). Locally transplanted human urine-induced nephron progenitor cells contribute to renal repair in mice kidney with diabetic nephropathy. Biochem. Biophys. Res. Commun..

[21] Yu S., Yu S., Liu H., Liao N., Liu X. (2023). Enhancing mesenchymal stem cell survival and homing capability to improve cell engraftment efficacy for liver diseases. Stem Cell Res. Ther..

[22] Scopes R.K., Scopes R.K. (1994). Optimization of Procedures; Final Steps. Protein Purification: Principles and Practice.

[23] Doonan S., Cutler P., Cutler P. (2004). General Strategies. Protein Purification Protocols.

[24] Walker S.J., Zhao S.Y., Orlando G., Remuzzi G., Williams D.F. (2017). Chapter 70—Markers of Repair and Regeneration in the Marginal Kidney. Kidney Transplantation, Bioengineering and Regeneration.

[25] Tsuji K., Kitamura S. (2015). Trophic Factors from Tissue Stem Cells for Renal Regeneration. Stem Cells Int..

[26] Zheng J., Park K., Jang J., Son D., Park J., Kim J., Yoo J.-E., You S., Kim I.-Y. (2024). Utilizing stem cell-secreted molecules as a versatile toolbox for skin regenerative medicine. J. Control. Release.

[27] Humphreys B.D., Valerius M.T., Kobayashi A., Mugford J.W., Soeung S., Duffield J.S., McMahon A.P., Bonventre J.V. (2008). Intrinsic epithelial cells repair the kidney after injury. Cell Stem Cell.

[28] Sung M.J., Kim D.H., Jung Y.J., Kang K.P., Lee A.S., Lee S., Kim W., Davaatseren M., Hwang J.-T., Kim H.-J. (2008). Genistein protects the kidney from cisplatin-induced injury. Kidney Int..

[29] Modaresi A., Nafar M., Sahraei Z. (2015). Oxidative stress in chronic kidney disease. Iran. J. Kidney Dis..

[30] Gyurászová M., Gurecká R., Bábíčková J., Tóthová Ľ. (2020). Oxidative Stress in the Pathophysiology of Kidney Disease: Implications for Noninvasive Monitoring and Identification of Biomarkers. Oxidative Med. Cell. Longev..

[31] Hosohata K. (2016). Role of Oxidative Stress in Drug-Induced Kidney Injury. Int. J. Mol. Sci..

[32] Saravanakumar K., Park S., Santosh S.S., Ganeshalingam A., Thiripuranathar G., Sathiyaseelan A., Vijayasarathy S., Swaminathan A., Priya V.V., Wang M.H. (2022). Application of hyaluronic acid in tissue engineering, regenerative medicine, and nanomedicine: A review. Int. J. Biol. Macromol..

[33] Furnus C.C., de Matos D.G., Martínez A.G. (1998). Effect of hyaluronic acid on development of in vitro produced bovine embryos. Theriogenology.

[34] Jin K., Hyun Kuk S., Hyun Wook L., Eun Young P., Wan Young K. (2006). Developmental Expression of Hyaluronan in Rat Kidney. Am. Soc. Nephrol. Natl. Meet..

[35] Kurisawa M., Chung J.E., Yang Y.Y., Gao S.J., Uyama H. (2005). Injectable biodegradable hydrogels composed of hyaluronic acid-tyramine conjugates for drug delivery and tissue engineering. Chem. Commun..

[36] Gilli R., Kacuráková M., Mathlouthi M., Navarini L., Paoletti S. (1994). FTIR studies of sodium hyaluronate and its oligomers in the amorphous solid phase and in aqueous solution. Carbohydr. Res..

[37] Abdallah M., Martin M., El Tahchi M.R., Balme S., Faour W.H., Varga B., Cloitre T., Páll O., Cuisinier F.J.G., Gergely C. (2019). Influence of Hydrolyzed Polyacrylamide Hydrogel Stiffness on Podocyte Morphology, Phenotype, and Mechanical Properties. ACS Appl. Mater. Interfaces.

[38] Melica M.E., La Regina G., Parri M., Peired A.J., Romagnani P., Lasagni L. (2019). Substrate Stiffness Modulates Renal Progenitor Cell Properties via a ROCK-Mediated Mechanotransduction Mechanism. Cells.

[39] Cherstvy A.G., Thapa S., Wagner C.E., Metzler R. (2019). Non-Gaussian, non-ergodic, and non-Fickian diffusion of tracers in mucin hydrogels. Soft Matter.

[40] Fujiyabu T., Li X., Chung U.-i., Sakai T. (2019). Diffusion Behavior of Water Molecules in Hydrogels with Controlled Network Structure. Macromolecules.

[41] Axpe E., Chan D., Offeddu G.S., Chang Y., Merida D., Hernandez H.L., Appel E.A. (2019). A Multiscale Model for Solute Diffusion in Hydrogels. Macromolecules.

[42] Tögel F., Zhang P., Hu Z., Westenfelder C. (2009). VEGF is a mediator of the renoprotective effects of multipotent marrow stromal cells in acute kidney injury. J. Cell. Mol. Med..

[43] Tsujimura T., Idei M., Yoshikawa M., Takase O., Hishikawa K. (2016). Roles and regulation of bone morphogenetic protein-7 in kidney development and diseases. World J. Stem Cells.

[44] Jooybar E., Abdekhodaie M.J., Alvi M., Mousavi A., Karperien M., Dijkstra P.J. (2019). An injectable platelet lysate-hyaluronic acid hydrogel supports cellular activities and induces chondrogenesis of encapsulated mesenchymal stem cells. Acta Biomater..

