# Supplementary material for: Hydrogel-Mediated Local Delivery of Induced Nephron Progenitor Cell-Sourced Molecules as a Cell-Free Approach for Acute Kidney Injury
Source: Int J Mol Sci. 2024 Oct 2;25(19):10615. doi: 10.3390/ijms251910615 (PMC11477367; doi:10.3390/ijms251910615)
Supplement: Supplementary file 1 [file ijms-25-10615-s001.zip › ijms-3208093-supplementary.pdf]

SUPPLEMENTARY DATA

Hydrogel-mediated local delivery of induced nephron progenitor cell-sourced molecules as a cell-free therapy for acute kidney injury

Kyoungmin Park [1, †], Wei-Wei Gao [1, †], Jie Zheng [1,4], Kyung Taek Oh [1], In Yong Kim [3,*], Seungkwon You [1,2,*]

[1] Department of Biotechnology, College of Life Sciences and Biotechnology, Korea University, Seoul 02841, Republic of Korea

[2] Institute of Animal Molecular Biotechnology, Korea University, Seoul 02841, Republic of Korea

[3] Catholic High-Performance Cell Therapy Center & Department of Medical Life Science, College of Medicine, The Catholic University of Korea, Seoul 06591, Republic of Korea

[4] Present Address: Wenzhou Institute, University of Chinese Academy of Sciences, Wenzhou, 325000, China

*Corresponding author: Seungkwon You (bioseung@korea.ac.kr) and In Yong Kim (inyongkim79@gmail.com)

Supplementary tables

Table S1. Primer information used in Real-time PCR

In vitro study		
**Gene**	**Full name**	**Primer sequences (5' to 3')**
**p53**	Tumor protein p53	CCTCAGCATCTTATCCGATGG<br>TGGATGGTGGTACAGTCAGAGC
**p21**	Cyclin-dependent kinase inhibitor 1	AGGTGGACCTGGAGACTCTCAG<br>TCCTCTTGGAGAAGATCAGCCG
**Caspase 3**	Caspase 3	GGAAGCGAATCAATGGACTCTGG<br>GCATCGACATCTGTACCAGACC
**Caspase 7**	Caspase 7	CGGAACAGACAAAGATGCCGAG<br>AGGCGGCATTTGTATGGTCCTC
**Bax**	Bcl2-associated X protein	TCAGGATGCGTCCACCAAGAAG<br>TGTGTCCACGGCGGCAATCATC
**Bcl2**	B-cell lymphoma 2	ATCGCCCTGTGGATGACTGAGT<br>GCCAGGAGAAATCAAACAGAGGC
**GAPDH**	Glyceraldehyde-3-phosphate dehydrogenase	GTCTCCTCTGACTTCAACAGCG<br>ACCACCCTGTTGCTGTAGCCAA
In vivo study		
**Gene**	**Full name**	**Primer sequences (5' to 3')**
**TNF-α**	Tumor necrosis factor alpha	AGCCCCCAGTCTGTATCCTT<br>CTCCCTTTGCAGAACTCAGG
**IL6**	Interleukin 6	AGACAGCCACTCACCTCTTCAG<br>TTCTGCCAGTGCCTCTTTGCTG
**BCL2**	B-cell lymphoma 2	CCCTTTCCTAGACCCAGCAA<br>AAACCCTCCATCCTGTCCAG
**FGF2**	Fibroblast growth factor 2	GCGACCCACACGTCAAACTA<br>TCCCTTGATAGACACAACTCCTC
**VEGF**	Vascular endothelial growth factor	TTTGCCAATCACACTTCCTGC<br>ACACTGTGGTAATGTTGCTGG
**BMP7**	Bone morphogenetic protein 7	ACCCTCGATACCACCATCGG<br>GCTCCCGGATGTAGTCCTT
**GAPDH**	Glyceraldehyde-3-phosphate dehydrogenase	TGTGTCCGTCGTGGATCTGA<br>CCTGCTTCACCACCTTCTTGA

Table S2. Antibody information used in Western blot

**Antibody**	**Dilution**	**Company**
Anti-p53	1:10000	Abcam
Anti-Caspase 3	1:5000	Abcam
Anti-Cleaved caspase 3	1:500	Abcam
Anti-Bax	1:1000	Abcam
Anti-α tubulin	1:1000	Santa Cruz

Supplementary figures

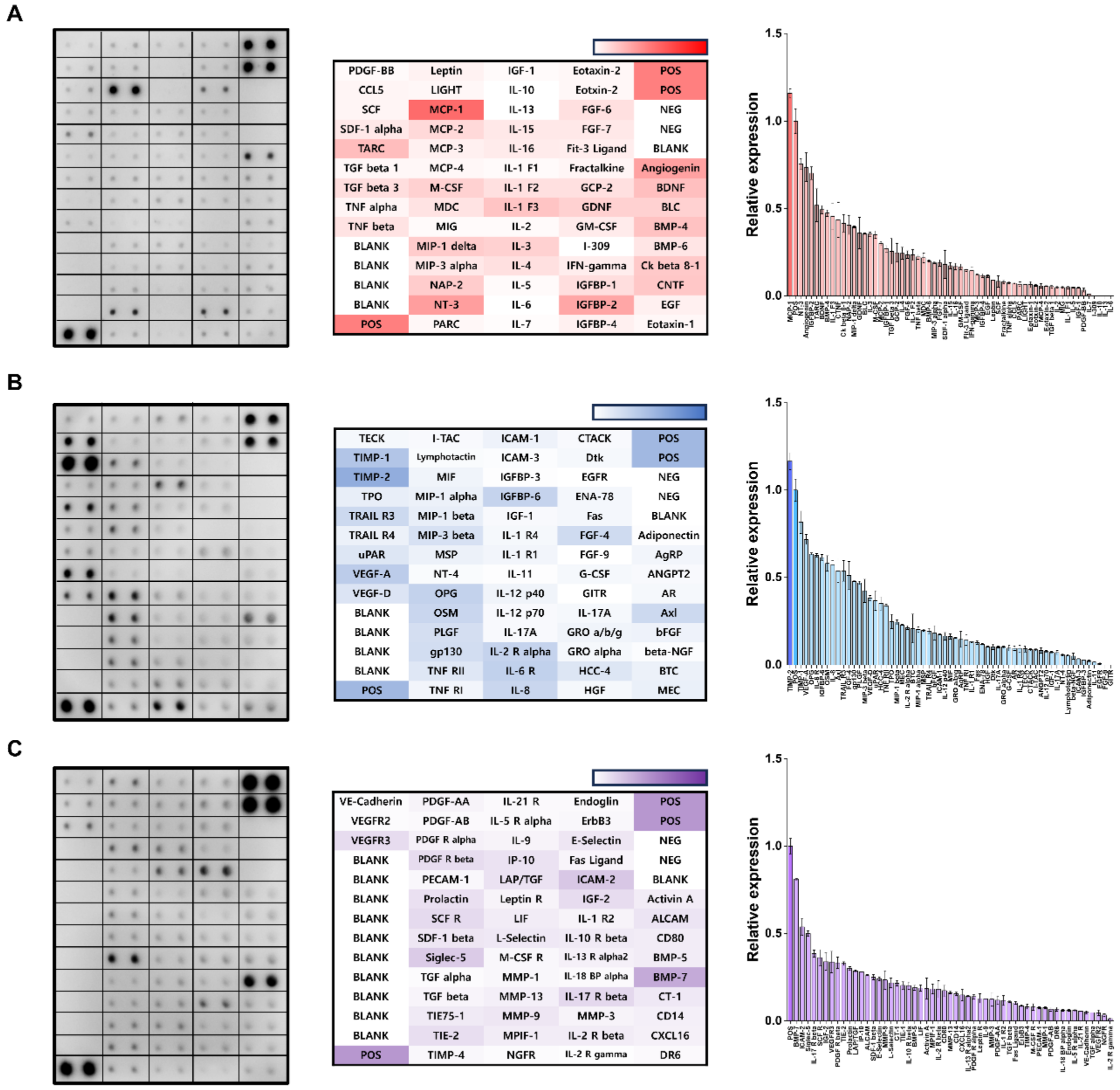

Figure S1. Antibody-based protein array of iNPC-SM.

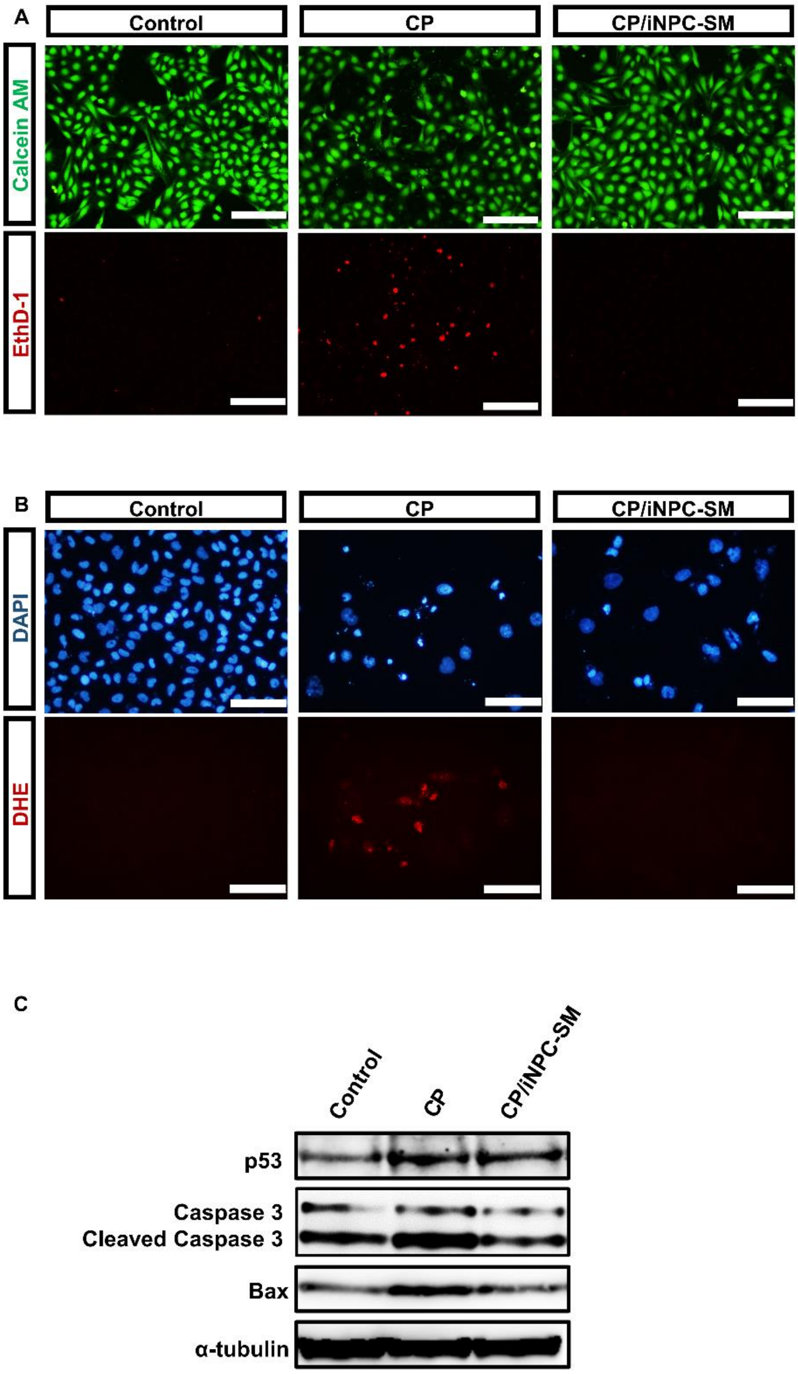

A
Control
CP
CP/iNPC-SM
Calcein AM
EthD-1
B
Control
CP
CP/iNPC-SM
DAPI
DHE
C
Control
CP
CP/iNPC-SM
p53
Caspase 3
Cleaved Caspase 3
Bax
α-tubulin

Figure S2. **(A)** Live/dead staining of HK2 cells treated with CP and CP/iNPC-SM. Scale bars, 200 µm. **(B)** DHE staining of HK2 cells treated with CP and CP/iNPC-SM. Nuclei were counterstained with DAPI. Scale bars, 200 µm. **(C)** Western blot images of apoptotic markers.

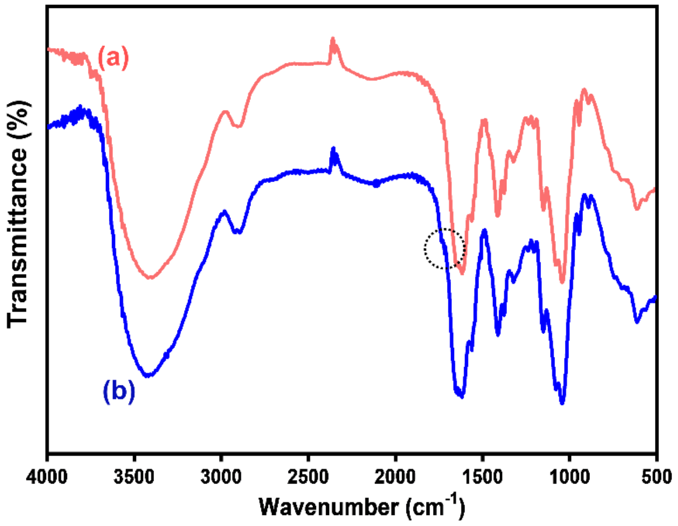

Figure S3. FT-IR of Hyaluronic acid (a) and HA-Tyr conjugate (b)
